# The green seaweed *Ulva*: a model system to study morphogenesis

**DOI:** 10.3389/fpls.2015.00072

**Published:** 2015-02-19

**Authors:** Thomas Wichard, Bénédicte Charrier, Frédéric Mineur, John H. Bothwell, Olivier De Clerck, Juliet C. Coates

**Affiliations:** ^1^Institute for Inorganic and Analytical Chemistry, Jena School for Microbial Communication, Friedrich Schiller University Jena, Jena, Germany; ^2^UMR 8227, Integrative Biology of Marine Models, Station Biologique de Roscoff, Centre National de la Recherche Scientifique, Roscoff, France; ^3^UMR 8227, Integrative Biology of Marine Models, Station Biologique de Roscoff, Sorbonne Universités, UPMC University of Paris 06, Roscoff, France; ^4^School of Biological Sciences, Queen’s University of Belfast, Belfast, UK; ^5^School of Biological and Biomedical Sciences and Durham Energy Institute, Durham University, Durham, UK; ^6^Phycology Research Group and Center for Molecular Phylogenetics and Evolution, Ghent University, Ghent, Belgium; ^7^School of Biosciences, University of Birmingham, Birmingham, UK

**Keywords:** algal genetics, chlorophyta, green tides, holobiont, multicellular organism, model organism

## Abstract

Green macroalgae, mostly represented by the Ulvophyceae, the main multicellular branch of the Chlorophyceae, constitute important primary producers of marine and brackish coastal ecosystems. *Ulva* or sea lettuce species are some of the most abundant representatives, being ubiquitous in coastal benthic communities around the world. Nonetheless the genus also remains largely understudied. This review highlights *Ulva* as an exciting novel model organism for studies of algal growth, development and morphogenesis as well as mutualistic interactions. The key reasons that *Ulva* is potentially such a good model system are: (i) patterns of *Ulva* development can drive ecologically important events, such as the increasing number of green tides observed worldwide as a result of eutrophication of coastal waters, (ii) *Ulva* growth is symbiotic, with proper development requiring close association with bacterial epiphytes, (iii) *Ulva* is extremely developmentally plastic, which can shed light on the transition from simple to complex multicellularity and (iv) *Ulva* will provide additional information about the evolution of the green lineage.

## INTRODUCTION

The marine seaweed *Ulva* belongs to the chlorophytes, an informal assemblage of three traditional classes (Ulvo-, Trebouxio- and Chlorophyceae) that evolved from unicellular marine planktonic prasinophyte algae in the Neoproterozoic (Herron et al., 2009; Verbruggen et al., 2009; Parfrey et al., 2011). Although recently considerable doubts have arisen regarding the monophyly of the three classes making up the core Chlorophytes (Zuccarello et al., 2009; Fucikova et al., 2014; Lemieux et al., 2014), for the sake of clarity we will refer to them by their traditional names, unless otherwise specified. The Chloro- and Trebouxiophyceae diversified largely in freshwater and terrestrial habitats, while Ulvophyceae came to dominate shallow marine environments (Becker and Marin, 2009). Ulvophyceae display an astounding morphological and cytological diversity (Cocquyt et al., 2010). This includes unicells, filaments, sheet-like thalli (vegetative shoot-like tissues) and giant-celled coenocytic or siphonal seaweeds (Mine et al., 2008; Cocquyt et al., 2010), which branch and fuse to form morphologies with root-, stem- and leaf-like structures comparable in size to large shrubs on land (Chisholm et al., 1996; Vroom and Smith, 2003; Littler et al., 2005).

**FIGURE 1:**
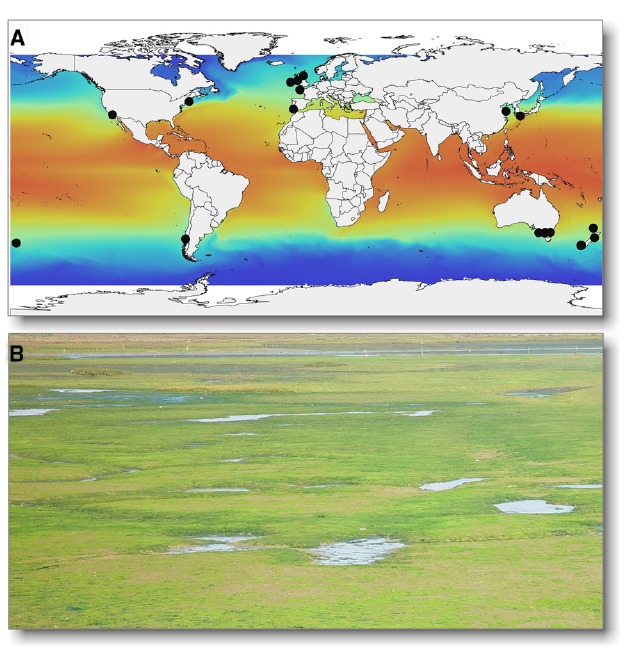
**(A)** The worldwide distribution of *U. compressa* and related populations including *U. mutabilis* (black circles; rbcL haplotypes available through NCBI GenBank) are presented as an example of the cosmopolitan nature of *Ulva* spp. The sea surface temperature map was plotted using Bio-ORACLE^1^ (Tyberghein et al., 2012). **(B)** *Ulva* can cause green tides, e.g., in the lagoon Ria Formosa (Portugal). Photo is a courtesy of Dr. Eric-Jan Malta (IFAPA, Spain).

The Ulvophyceae thus form an excellent group of organisms in which to elucidate the evolutionary processes and genetic mechanisms underlying morphological diversity, which is also influenced by associated bacteria *via* cross-kingdom cross-talk. Within the order Ulvales, where all species have uninucleate cells, algae present simple morphologies (Brodie et al., 2007). The Ulvaceae stand out by their organization into a basic “diptych” plan, either tubular thalli (e.g., *Blidingia*) or flattened distromatic (2 cells thick) blades (e.g., *Umbraulva*). Both morphological forms are present in the genus *Ulva* [“enteromorpha” (tubular) and “sea lettuces” (flattened)], appearing concomitantly in many sub-clades reported in molecular phylogenies of the genus (Hayden and Waaland, 2002; Hayden et al., 2003), including at the species level (e.g., *Ulva mutabilis* and *U. compressa*; Løvlie, 1964; Tan et al., 1999; see Figure 1A).

From an economic perspective, green seaweeds are sustainable biomass feedstocks for the food and biotech industries, including bioremediation, integrated aquaculture systems and potential biofuel production (Nisizawa et al., 1987; Neori et al., 1996, 2004; Dibenedetto, 2012; Alsufyani et al., 2014). *Ulva* is increasingly important in coastal ecosystem management, due to eutrophication-driven green tides in shallow environments (Figure 1B; Leliaert et al., 2009; Teichberg et al., 2010; Gosch et al., 2012; Smetacek and Zingone, 2013).

The aim of this review is to summarize key features of *Ulva*, to stress understudied fundamental questions in algal developmental biology, and to highlight new perspectives on the “*Ulva* genetic tool kit.”

## REGULATION AND MANIPULATION OF THE LIFE CYCLE

Although summarized as a simple alternation of isomorphic generations, the “haplodiplontic” life-cycle of many *Ulva* species is generally more complex (Føyn, 1958; Hoxmark, 1975; Phillips, 1990) and has been extensively investigated in *U. lactuca* and *U. mutabilis*. The two macroscopic stages, the sporophyte and gametophyte, can each originate in more than one way. Diploid multicellular sporophytes can originate from the fusion of two gametes of opposite mating type. Haploid gametophytes can derive from meiotically-formed haploid zoids or from unmated biflagellated gametes. In addition, diploid parthenosporophytes can originate from unmated gametes by spontaneous diploidization and give raise to zoids of only one mating type, which develop into new gametophytes (Hoxmark, 1975; Fjeld and Løvlie, 1976).

Many seaweeds exhibit photoperiodic control over the reproduction of germ cells. This was first elucidated in *Ulva pseudocurvata*, which exhibited weekly peaks of gametophytic reproduction during the summer season (Lüning et al., 2008). Conversely, in *U. mutabilis*, sporulation inhibitors (SI) and a swarming inhibitor (SWI) have been identified, respectively controlling the gametogenesis and subsequent gamete release, independently of photoperiod (Nilsen and Nordby, 1975; Stratmann et al., 1996; Wichard and Oertel, 2010). Vegetative thalli release a high molecular mass cell wall glycoprotein (SI-1) into the surrounding medium while containing a second low molecular weight inhibitor (SI-2) in the space between the two cell layers of the thallus. The transformation from a blade cell into a gametangium occurs only if SI-1 levels drop and the constantly-present SI-2 is no longer perceived by the alga, as discussed by Stratmann et al. (1996).

Facilitating the potential of *Ulva* as a model organism, gametogenesis can be induced artificially by removal of both SI, *via* cutting the thallus into single-layer fragments and subsequently washing, as exemplified originally for *U. mutabilis* by Stratmann et al. (1996), but also observed in *U. lactuca*, *U. linza*, and *U. rigida* (Stratmann et al., 1996; Wichard and Oertel, 2010; Vesty et al., 2015). After induction, gametes are released by removal of the SWI (accumulated during gametogenesis) synchronizing the discharge of the gametangia and increasing the mating probability (Wichard and Oertel, 2010). Moreover, unmated gametes can develop parthenogenetically into clonal, haploid gametophytes ideal for genetic manipulation and reproducible standardized experiments. The generation time of *U. mutabilis* is short: only 3–5 weeks’ growth is required between potential inducibility of synchronous gametogenesis (Løvlie, 1964; Stratmann et al., 1996).

The sporulation-inhibitor-regulated life-cycle transition may have strong relevance to the dynamics of green tide formations, as fragmentation is often pivotal during algal bloom succession (Gao et al., 2010).

## SYMBIOTIC NATURE OF *Ulva* GROWTH

Cross-kingdom cross-talk between macroalgae and bacteria controls algal settlement, growth and development (Joint et al., 2002, 2007). Several studies have shown that *Ulva* fails to form its typical morphology in the absence of the appropriate bacteria and simply proliferates as an undifferentiated clump of callus cells (e.g., Fries, 1975; Marshall et al., 2006; Spoerner et al., 2012).

Interactions between *Ulva* spp. and their associated bacteria have been well-characterized over the last 50 years and the bacterial colonization of *Ulva* species has been defined based on 16S rDNA gene phylogeny (Burke et al., 2009; Lachnit et al., 2009). Burke et al. (2011a) showed that the algal microbiota of *U. australis* varies over the season, and between very close sample sites. Although they did not rigorously verify the mono-specificity of their *Ulva* samples, they concluded that *Ulva* does not possess a core microbial community but that the assemblage of epibacteria is determined by a “lottery” rather than controlled by mechanistic (e.g., mutualistic) interactions (Tujula et al., 2010; Burke et al., 2011a,b). Even though this may be true for a large part of the associated bacterial community, specific bacteria, essential for settlement, growth and morphogenesis, have been isolated consistently from *Ulva* species, indicating that sometimes “hard-to-find bacteria” can harbor essential eco-physiological functions in the host-microbe system (Spoerner et al., 2012). A specific selection mechanism was suggested for *Ulva* zoospore settlement, which occurs preferentially on bacteria-colonized surfaces. The numbers of attached zoospores were proportional to the size of the bacterial population, which releases N-acyl-homoserine lactones (AHLs; Tait et al., 2005, 2009; Joint et al., 2007).

**FIGURE 2:**
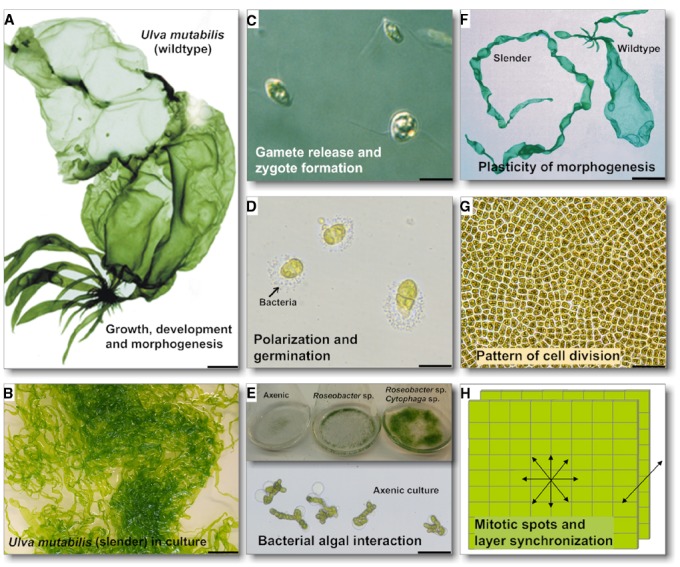
**Characteristics of *Ulva*, and scientific contributions to research into *Ulva* growth, development and morphogenesis.** Key events in *Ulva* ontogenesis are highlighted: **(A,B)** The wildtype *U. mutabilis* and a typical culture of its developmental mutant *slender* are shown (scale bar = 1 cm). Regulation of gametogenesis/zoosporogenesis, gamete/zoospore release and zygote formation are important checkpoints in the isomorphic, haplodiplontic *Ulva* life cycle. The wildtype **(A)** released gametes (**C,** scale bar = 5 μm) upon synchronous sporulation in the apical part of the thallus (= discharged colorless gametangia). **(D)** Unmated gametes propagate as a haploid strain and germinate with a clear polarization for primary rhizoid formation upon settlement, where bacteria that accumulate can be observed (biofilm formation). **(E)** Under axenic conditions *Ulva* develops into a callus with no cell differentiation and slow growth (1 week old culture; scale bar = 50 μm). However, morphogenesis can be recovered by a combination of two essential bacteria releasing morphogenetic compounds into the growth medium (3 weeks old culture). **(F)** Several *U. mutabilis* developmental mutants have been found: the fast-growing developmental mutant *slender* shows only traces of the sea lettuce like morphotype of the wildtype (scale bar = 1 cm). **(G,H)** It is hypothesized that mitotic spots spread over the thallus cause potential irregular extensions of the blade sheet, and that bilayer formation is regulated. These key questions must be addressed in future studies (scale bar in G = 50 μm). Images of *U. mutabilis* **(A)** and of gametes **(C)** were reprinted from Wichard and Oertel (2010) with permission from John Wiley and Sons, Copyright © (2010) Wiley. Image of the *Ulva* thallus **(G)** is a courtesy of Dr. D. Saint-Marcoux (University of Oxford, UK).

As bacteria are essential for normal green seaweed development it is tempting to assume that AHL signaling may initiate the cross-kingdom cross-talk. Several studies using axenic cultures demonstrated that bacterial factors control growth, development and/or morphogenesis of Ulvales, e.g., in *Ulva* and *Monostroma* (Provasoli, 1958; Fries, 1975; Bonneau, 1977; Provasoli and Pintner, 1980; Nakanishi et al., 1996; Matsuo et al., 2005; Marshall et al., 2006; Singh et al., 2011; Spoerner et al., 2012). A single isolated bacterial strain could not completely restore normal development of antibiotic-treated axenic *U. linza* into mature thalli (Marshall et al., 2006), indicating potential synergistic effects of bacteria on thallus development. Spoerner et al. (2012) separated discharged gametes from their accompanying bacteria by taking advantage of the gametes’ fast movement toward light. Axenic *U. mutabilis* gametes develop into callus-like colonies composed of undifferentiated cells with malformed cell walls (Figure 2). Complete morphogenesis was recovered by a combination of two bacterial strains, *Roseobacter* sp. and *Cytophaga* sp., or by morphogenetic compounds extracted from both bacterial supernatants (Spoerner et al., 2012). The *Roseobacter* species exhibits a specific chemotactic affinity to the rhizoid cells of *U. mutabilis* (Figure 2) and seems to cooperate with the *Cytophaga* strain and the alga by chemical communication, forming a symbiotic tripartite community. Here, *Roseobacter* sp. and *Cytophaga* sp. fulfill a complementary task: *Roseobacter* sp. induces cell division similar to a cytokinin, whereas the *Cytophaga* sp. factor, similar to auxin, induces the basal stem cell and primary rhizoid cells, which form the algal holdfast. Interestingly, whereas the *Roseobacter* can be replaced with other α-proteobacteria (including *Sulfitobacter* sp.) and γ-proteobacteria (*Halomonas* sp.), the presence of the *Cytophaga* sp. seems to be mandatory, suggesting that potentially specific genes drive the community of *Ulva* and its associated bacteria (Spoerner et al., 2012).

Importantly, bacteria can induce algal development and morphogenesis when separated from axenic gametes by a membrane (Spoerner et al., 2012), implying that diffusible morphogenetic compounds are released into the seawater and become part of the chemosphere of the alga and its associated bacteria. Indeed, several morphogenesis-inducing substances have been (partly) purified from bacterial extracts (Matsuo et al., 2005; Spoerner et al., 2012). To date, only Matsuo et al. (2005) have elucidated the structure of a morphogenetic compound, named “thallusin,” isolated from a bacterium of the *Cytophaga*-Flavobacterium-Bacteroides group, which restores the foliaceous morphology of *Gayralia oxyspermum* (previously *Monostroma oxyspermum*). Unlike for other macroalgae that interact with bacteria (Goecke et al., 2010), axenic *Ulva* gametes are easily-obtainable and can be subsequently inoculated with key morphogenesis-inducing bacteria/factors, facilitating reproducible and standardized experimental conditions (Spoerner et al., 2012; Wichard, 2015). The early-developmental processes of germination and cell differentiation, and subsequent phenotypic plasticity, can be now investigated either under axenic conditions or within a defined microbial community.

## GROWTH PATTERNS AND PLASTICITY OF MORPHOGENESIS

*Ulva* has a relatively simple multicellular organization that can give rise, through phenotypic plasticity, to a range of moderately complex morphologies. The thallus contains three cell types (rhizoid, stem and blade cells) that divide synchronously under standardized conditions once a day as demonstrated for *U. mutabilis* (Løvlie, 1978; Stratmann et al., 1996).

In some species growth occurs by co-ordinated successive mitoses in parallel cell files (e.g., *U. linza*), while in others (e.g., *U. fasciata*, *U. taeniata*, *U. australis*) extension of the blade occurs irregularly, e.g., *via* local mitotic spots spread over the thallus (Figure 2). This raises fundamental questions about *Ulva* developmental biology. Mitotic and cell-differentiation activities could be controlled by long-range diffusible agents obeying Turing’s reaction-diffusion chemical models (Torii, 2012), as indicated by the inducibility of gametogenesis along the longitudinal axis of the thallus, from the apex to the rhizoid (Wichard and Oertel, 2010). However, the mitotic potential might also be transmitted to the daughter cells through inheritance of a transcription factor during asymmetric cell division, as in *Arabidopsis stomata* guard cell precursors (Robinson et al., 2011). In addition, the role of the extracellular matrix within the thallus for cell determination and differentiation might be particularly intriguing (Fjeld and Løvlie, 1976).

Mechanical constraints generated by neighboring growth could affect mitotic activities, as observed in the green alga *Coleochaete orbicularis* (Coleochaetales), where computer simulation showed that polarized cell growth primarily depends on the size and shape of the neighboring cells (Dupuy et al., 2010). The different growth mode of these two green algae-through autonomous cell files growing centrifugally for *C. orbicularis* (Marchant, 1974) and potentially through local mitotic spots for example in *Ulva fasciata* (B.C., personal observation)-encourage further comparison between these two systems.

The superimposition of cell layers with different cell fates is common in multicellular organisms. The coordination between the two *Ulva* thallus cell layers is not understood, but represents a much simpler system to study this process than, for example, an angiosperm leaf. Initially, a change in cell polarity must have occurred to allow the formation of two layers, followed by parallel expansion of each layer. Understanding whether the two cell layers act autonomously and independently from each other can be determined by local ablation of a single cell layer, or by clonal analysis to follow specific cell lineages during growth *via* selection for *Ulva* individuals displaying mosaic sectors (e.g., transposon-mediated GUS expression, Lee et al., 1995; Lemieux et al., 2014) or mutations (Vincent et al., 1995; Scanlon, 2000). Within the monophyletic *Ulva*/*Enteromorpha* grouping, there are several cryptic clades including the so-called *pseudocurvata*/*compressa* group that could not be detected based on morphology alone, but required molecular data for its identification (Tan et al., 1999). Molecular phylogeny showed that changes in gross morphological characteristics, e.g., the switch from monostromatic tubes to distromatic sheets, have occurred several times during evolution, but infrequently, as the phylogenetic tree does not show many mixed clades of *Ulva* and *Enteromorpha* (Tan et al., 1999). Of particular interest was *U. mutabilis* (Føyn, 1958), because it repeatedly gave rise to morphological mutants under laboratory conditions. Føyn and co-workers described several of the observed mutants, named e.g., *slender*, *long*, *branched*, *lumpy*, or *bubble* (Bryhni, 1974; Fjeld and Børresen, 1975). The fast growing mutant *slender* is one of the most interesting variations, which shows only traces of the sea lettuce-like morphotype of the wildtype (Figure 2; Slender versus wildtype). As many developmental mutants such as *lumpy* show disoriented division planes along with defects in cell wall production, genetic control of morphogenesis (i.e., whether a particular developmental program is activated), may also depend on the biochemical machinery of cell wall biosynthesis (Bryhni, 1974; Fjeld and Løvlie, 1976). Due to its mutational instability, even 50 years ago, Løvlie proposed *U. mutabilis* Føyn as a good green multicellular model organism for analyses of the genetic control of cell division and morphogenesis (Løvlie, 1964, 1968). Interestingly, the bacteria necessary for complete morphogenesis (see Symbiotic Nature of *Ulva* Growth), do not influence the algal morphotype (Spoerner et al., 2012). The underlying molecular mechanism of radical changes in morphology needs further investigation preferably by utilizing a working genetic system.

Overall, the described standardized culture conditions (see Symbiotic Nature of *Ulva* Growth) as well as the production of large numbers of unicells and isomorphic haploid and diploid structures (as outlined by Coates et al., 2014) are unique to *Ulva*. Developmental processes can be thus easily studied including, e.g., germination, cell adhesion, cell differentiation and morphogenesis. Ultimately, these studies will help to understand the morphological evolution from uni- to multicellularity in green plants.

## PRESENT AND FUTURE *Ulva* “GENETIC TOOL KIT”

A model organism for multicellular marine Chlorophyte algae has been lacking to date, largely due to the difficulty of working with these organisms in laboratory cultures. Their dependence on signals from epiphytic micro-organisms to assume their correct development and morphology makes them quite different to well established models such as *Arabidopsis*, *C. elegans* and *Drosophila*. The recent breakthroughs in understanding *Ulva*-bacterial interactions and developing axenic culture methods (Patel et al., 2003; Marshall et al., 2006; Wichard and Oertel, 2010; Spoerner et al., 2012) mean that *U. mutabilis* now represents an excellent candidate to fill this research gap. *U. mutabilis* combines a short and controllable life cycle with simple morphology, emerging genetics, a small genome (∼100 Mbp) and prolific spore/gamete production. Moreover, it is closely-related to species of economic and ecological importance.

The development of *U. mutabilis* as a model organism is particularly timely given the falling costs of large-scale DNA and RNA sequencing due to advances in high-throughput sequencing technologies. What is currently missing for *Ulva* to become a fully useful model is the availability of extensive large-scale genomic data. Some *U. linza* ESTs have been produced (Stanley et al., 2005), and a partial *U. linza* transcriptome (RNA-seq data) has been published (Zhang et al., 2012). A partial transcriptome and cDNA/EST library have been generated for *U. prolifera*, the green tide-forming alga in the Chinese Yellow Sea (Li et al., 2012; Xu et al., 2012) and small RNAs have been characterized by high-throughput sequencing (Huang et al., 2011). Moreover, reference genes for *Ulva* RT-PCR studies have been identified (Dong et al., 2012).

A project to sequence the genome of *U. mutabilis* has been initiated (funded by the UK Natural Environment Research Council), highlighting the perceived importance of defining the *Ulva* genetic toolkit. Hand-in-hand with this should come deeper transcriptome analyses, which will aid genome assembly. Such RNA-seq experiments can also provide essential information about profiles of gene expression during the *Ulva* life cycle and development, without the requirement for making *Ulva* microarrays. Only with these kinds of data in place we can begin to fully understand the molecular mechanisms controlling green seaweed morphogenesis, and compare them with the developmental mechanisms used by land plants (*Arabidopsis*, *Physcomitrella*) and other algae such as *Chlamydomonas*, *Volvox*, and *Ectocarpus*. In addition, comparative genomics of, e.g., the wildtype strain with the developmental mutant *slender* may help to identify genes that give each strain its unique characteristics.

The generation of transgenic lines will be one of the most powerful tools to develop for *Ulva*. This will enable tracking of *Ulva* cell lineages. Promoter-trap lines combining a non-targeted endogenous promoter with, e.g., a fluorescent marker green fluorescent protein (GFP), inserted in random positions in the *Ulva* genome, will allow for the identification of such cell lineages, as in *Arabidopsis* or in rice (Johnson et al., 2005; Kurup et al., 2005; Laplaze et al., 2005). Such transgenic lines will also both generate morphological mutants and allow the identification of the responsible genes (by inverse-PCR). The extensive success of the reporter-line approach in *Arabidopsis* since the 1990s (e.g., to identify cryptic promoters/enhancers) demonstrates how much our understanding of *Ulva* multicellularity should gain from our capability to genetically transform it (Topping et al., 1994; Pratibha et al., 2013). There are a few preliminary reports of stable transformation of Ulvales and other macroalgae (Mikami, 2013, 2014). Excitingly, a transformation vector has now been constructed for the preparation of a series of *E. coli*–*U. mutabilis* shuttle vector plasmids based on the bleomycin resistance gene (*ble*) and the expression signals of the chromosomal *rbcS* gene (Rubisco) from *U. mutabilis* (GenBank: EU176859.1). Transformation systems have been developed, including special vector plasmids for the introduction and expression of foreign genes in *Ulva*, for insertional mutagenesis, for gene tagging by plasmid integration into the genome, for protein-tagging by the GFP (GenBank: EU196041.1), and for cosmid cloning to prepare genomic gene-libraries for mutant gene complementation (W. Oertel, T. Wichard, A. Weissgerber, personal communication). The *Ulva* genetic toolkit will enable the field of green macroalgal molecular genetics to take off, with generation of (non-site directed) gene knock-outs, ectopic- and over-expression of genes, and cross-complementation studies between plants and seaweeds.

*Ulva* also provides a unique platform to study microbiomes: the requirement for associated bacteria to complete morphogenesis opens up the possibility for novel co-expression analyses of algal and bacterial gene expression during the life cycle, and perhaps integration with metabolomic data (as in Ritter et al., 2014), which will lead to a completely novel understanding of the algal-bacterial interactions controlling seaweed development. Tools for this kind of large-scale analysis are being developed at a rapid rate (McGettigan, 2013), due in part to human biology projects such as EnCODE and the gut microbiome but also explorative exo-metabolomic studies of the chemosphere of *Ulva* and the bacteria, which have been already successfully established (Alsufyani, 2014).

## CONCLUSION

Representative model organisms have been long-established in several major clades of plants, including the widely-used eudicot *Arabidopsis* and the monocot *Oryza*, for fundamental molecular genetic studies. The moss *Physcomitrella*, a non-vascular plant, is a favorable model for studies on the molecular evolution of plant development, while analysis of the *Chlamydomonas* genome has revealed the evolution of key plant functions (Merchant et al., 2007; Rensing et al., 2008). Green seaweed research will benefit hugely from the development of a model organism. In the last few decades, progress has been made in almost all areas of *Ulva* biology as summarized in this mini-review, including phylogenetics, biofouling, biofilm formation, biotic- and abiotic interactions, life-cycle regulation and genetics. *Ulva* is a promising model to understand tissue morphology and complex multicellularity and will improve our understanding of integrated and coordinated development. Learning from a well-established model organism will help to explore the biotechnology potential of *Ulva* and other seaweeds, e.g., in biomass and biofuel production. It also contributes to the understanding how the ecological resilience of an ecosystem depends upon both algae and microbiomes.

Until now, *Ulva* species were largely selected for scientific study based on their local abundance and ecological relevance. However, only a few *Ulva* species may fulfil the criteria for a model organism. Major factors that contribute to the usefulness of model organisms include standardized culture conditions, short life cycle, axenic cultures and the utilization of genetic analysis. *U. mutabilis* Føyn and its spontaneous available mutants possess an extremely short life cycle, and can be easily cultured along with its essential symbiotic bacteria to complete morphogenesis in a defined tripartite community. In addition, mass mating, availability of axenic cultures and the stable parthenogenetic propagation as a haploid strain will make the developmental mutant *slender* a particularly valuable model system for (evolutionary) developmental biology.

Orchestrated *Ulva* projects that unify scientists from various disciplines, for example, the newly-funded EU COST Action “*Advancing knowledge on seaweed growth and development*^2^,” and the PHYCOMORPH network^3^ now aim to unravel the rules that underpin the multiple interactions between an organism’s environment, genes, development and morphogenesis and to incorporate these rules into an evolutionary theory about the transition from simple to complex multicellularity.

### CONFLICT OF INTEREST STATEMENT

The authors declare that the research was conducted in the absence of any commercial or financial relationships that could be construed as a potential conflict of interest.

## Footnotes

1 http://www.oracle.ugent.be/

2 http://www.cost.eu/COST_Actions/fa/Actions/FA1406

3 www.phycomorph.org
